# Use of triiodothyronine to treat critically ill COVID-19 patients: a new clinical trial

**DOI:** 10.1186/s13054-020-02934-2

**Published:** 2020-05-08

**Authors:** Constantinos Pantos, Ioulia Tseti, Iordanis Mourouzis

**Affiliations:** Department of Pharmacology, Medical School, National and Kapodistrian University of Athens, 75 Mikras Asias Ave.,11527 Goudi, Athens, Greece

## Letter

As COVID-19 disease spreads, the number of critically ill patients requiring intensive care unit (ICU) support increases and mortality is remarkably high, calling for novel effective therapies to treat sepsis and septic shock in COVID-19 patients.

**Fig. 1:**
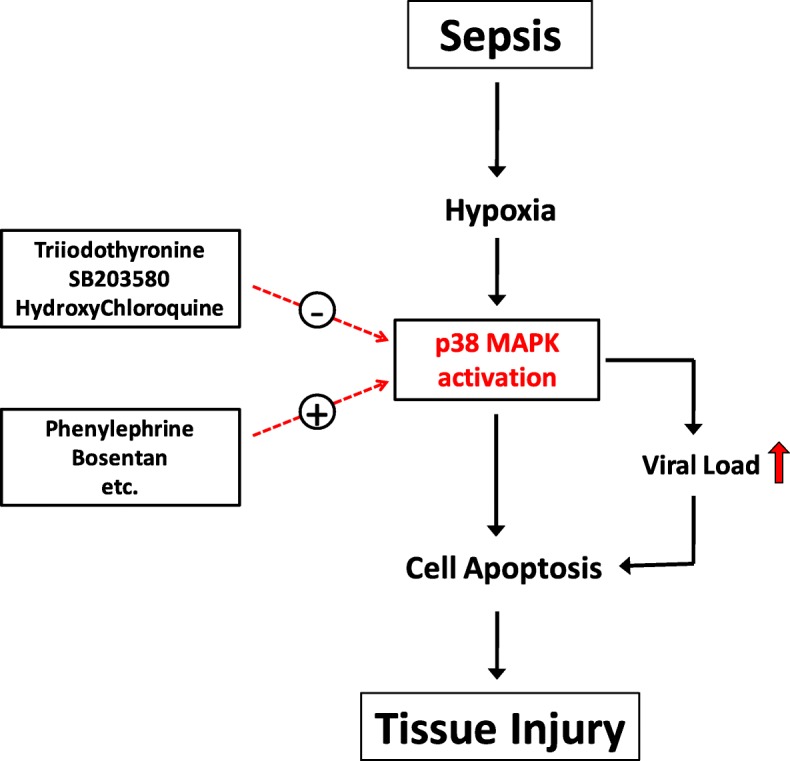
Proposed scheme showing that hypoxia and viral infectivity share common pro-apoptotic signaling pathways, such as p38 MAPK

From the pathophysiologist’s view, sepsis in viral infected patients results in cell damage due to both uncontrolled viral entry/replication and hypoxia. Hypoxia and viral infectivity share common pro-apoptotic signaling pathways, such as p38 mitogen-activated protein kinase (p38 MAPK) [1, 2] Fig. 1. In addition, inotropes and vasoactive agents, often used in ICU treatments, can increase tissue injury and viral load via p38 MAPK activation [1, 2]. Therefore, a vicious cycle is established in viral sepsis leading to high mortality despite the current therapies. Epinephrine and dobutamine use in sepsis is reported to be associated with increased mortality and atrial fibrillation [3].

Under acute illness, such as sepsis, myocardial infarction, and trauma, deregulation of thyroid hormone (TH) metabolism (non-thyroidal illness syndrome, NTIS) occurs and low circulating triiodothyronine (T3) is associated with increased mortality [4]. NTIS is of physiological relevance in cell defense, and it is now recognized that TH can increase tolerance of the cell to hypoxia via suppression of p38 MAPK activation and promote tissue repair through controlled Akt activation [1]. This novel reparative action of TH is currently investigated in the ThyRepair trial (EudraCT: 2016-000631-40) in patients with anterior STEMI undergoing angioplasty. In this study (almost completed), no major adverse effects have been observed.

TH also appears to prevent herpes simplex virus infectivity and potentiate host defense by increasing natural killer cells (NK) and enhancing the stimulatory effect of interferon on NK cells [5]. On the contrary, corticosteroids which are often used in ICU therapies suppress lung inflammation but also inhibit immune response and pathogen clearance and their use remains questionable.

On the basis of this evidence, a new phase II randomized, double blind, placebo controlled trial (Thy-Support, ClinicalTrials.gov Identifier: NCT04348513) is going to investigate the effect of intravenous high dose T3 (Uni-Pharma S.A., Greece) for enhancing recovery of critically ill COVID-19 patients.

## Data Availability

Not applicable

## References

[1] Pantos C, Mourouzis I (2015). Translating thyroid hormone effects into clinical practice: the relevance of thyroid hormone receptor α1 in cardiac repair. Heart Fail Rev.

[2] Marchant D, Dou Y, Luo H, Garmaroudi FS, McDonough JE, Si X (2009). Bosentan enhances viral load via endothelin-1 receptor type-A-mediated p38 mitogen-activated protein kinase activation while improving cardiac function during coxsackievirus-induced myocarditis. Circ Res.

[3] Sato R, Ariyoshi N, Hasegawa D, Crossey E, Hamahata N, Ishihara T, et al. Effects of inotropes on the mortality in patients with septic shock. J Intensive Care Med 2019:885066619892218. doi: 10.1177/0885066619892218. 10.1177/0885066619892218 31793373

[4] Padhi R, Kabi S, Panda BN, Jagati S (2018). Prognostic significance of nonthyroidal illness syndrome in critically ill adult patients with sepsis. Int J Crit Illn Inj Sci.

[5] Varedi M, Moattari A, Amirghofran Z, Karamizadeh Z, Feizi H (2014). Effects of hypo- and hyperthyroid states on herpes simplex virus infectivity in the rat. Endocr Res.

